# Promoting CHANGE cluster randomised controlled trial to improve food outlet healthiness in Australian sport and recreation facilities: protocol

**DOI:** 10.1136/bmjopen-2025-109584

**Published:** 2026-03-11

**Authors:** Neha Lalchandani, Helena Romaniuk, Adrian Cameron, Liliana Orellana, Jaithri Ananthapavan, A Peeters, Bettina Backman, Megan Adam, Steven Allender, Phuong Nguyen, Gary Sacks, Julie Kay Brimblecombe, Emma McMahon, Miranda Blake

**Affiliations:** 1 Institute for Health Transformation, Global Centre for Preventive Health and Nutrition, School of Health and Social Development, Deakin University, Melbourne, Victoria, Australia; 2 Biostatistics Unit, Faculty of Health, Deakin University, Melbourne, Victoria, Australia; 3 School of Public Health, The University of Queensland, Brisbane, Queensland, Australia; 4 Deakin Health Economics, Deakin University, Burwood, Victoria, Australia; 5 Deakin University, Melbourne, Victoria, Australia; 6 School of Exercise and Nutrition Sciences, Deakin University Faculty of Health, Burwood, Victoria, Australia; 7 Department of Nutrition, Dietetics and Food, Monash University, Clayton, Victoria, Australia; 8 Wellbeing and Preventable Chronic Disease Division, Charles Darwin University, Menzies School of Health Research, Darwin, Australian Capital Territory, Australia

**Keywords:** Randomized Controlled Trial, PUBLIC HEALTH, Implementation Science, HEALTH ECONOMICS, Protocols & guidelines

## Abstract

**Introduction:**

Food retail outlets in sports and recreation facilities often fail to support healthy eating, despite aligning with healthy lifestyles and goals of local governments (LGs) that often own or manage them. LGs face barriers to implementing facility changes including inadequate staffing, training and incentives. The Promoting CHANGE initiative was co-designed to support LGs in improving and sustaining healthier food and drink offerings in these settings.

**Methods and analysis:**

A 3-year, type 2 effectiveness-implementation hybrid cluster randomised controlled trial will evaluate the Promoting CHANGE capacity-building and support package in three Intervention and four Control LGs in Victoria, Australia (August 2023–July 2026). The co-designed initiative includes human resource support, training, tools, technical assistance, community-of-practice groups, feedback based on food outlet audit and sales data and small grant incentives. Using the RE-AIM (Reach, Effectiveness, Adoption, Implementation, Maintenance) evaluation framework, the trial’s co-primary outcomes are the percentage of least healthiest food and drinks (1) displayed (implementation) and (2) sold weekly (effectiveness). Key secondary outcomes are effectiveness (sales and revenue); facility-level adoption, implementation, maintenance of healthy changes; cost-effectiveness (within-trial modelled economic evaluation). Findings will provide evidence of the initiative’s effectiveness and scalability, informing recommendations for advancing healthier food environments in over 6000 community-based food outlets across 500 Australian LGs, with implications globally.

**Ethics and dissemination:**

This study has received approval from the Deakin University Human Research Ethics Committee (reference number HEAG-H 92_2023). The results will be published in scientific peer-reviewed journals along with plain language summaries for participants.

**Trial registration number:**

ACTRN12621001120864.

**STRENGTHS AND LIMITATIONS OF THIS STUDY**

- The Promoting CHANGE support package was co-designed with project partners using theory-informed and evidence-based implementation approaches, enhancing relevance and feasibility in real-world settings.
- The study employs a rigorous cluster randomised controlled trial using a type 2 hybrid design, allowing for simultaneous evaluation of intervention effectiveness and implementation processes.
- Integration of comprehensive six monthly audits and sales tracking provides robust, objective data on retail environment changes over time.
- Embedded economic and process evaluations strengthen the methodological framework by enabling in-depth analysis of implementation fidelity, costs and contextual factors.
- The potential for selection bias exists, as local governments with a pre-existing interest in nutrition may be more inclined to participate, which could limit generalisability.

## Introduction

 The health and economic burden of diet-related conditions such as obesity, type 2 diabetes and cardiovascular diseases underscores the urgent need to address unhealthy dietary consumption patterns in Australia^1^ and globally.^2^ The WHO recognises the pivotal role of physical, economic, political and socio-cultural surroundings in shaping dietary behaviours^3 4^ (collectively known as ‘food environments’^5^). In Australia, food retail outlets (henceforth ‘food outlets’) within sports and recreation facilities (henceforth ‘facilities’), such as cafés and kiosks (smaller, temporary or semi-permanent establishments), are often owned or managed by local governments (LGs). The role of LGs in advancing public health through localised action, including promoting healthier food retail environments,^3 6 7^ aligns with national strategies for obesity prevention and state-level and territory-level mandates.^8 9^ LGs can foster healthier food environments by implementing policies that enhance the availability and promotion of nutritious food and drink options,^10 11^ encouraging healthy sponsorships and partnerships and supporting health promotion initiatives for community well-being.^12 13^ Customer surveys have shown strong public support for providing healthier food and drink offerings in Australian LG settings,^14^,^16^ while council managers have expressed increased optimism in the value of these strategies following the implementation of a healthy beverage policy.^14^ However, despite the association of sports and recreation settings with healthy lifestyles, these facilities often do not support healthy eating.^17 18^ This paradox highlights the need for targeted interventions to transform these spaces into environments that align with public health objectives.

There is strong evidence that the healthiness of customer purchases can be improved by addressing the ‘4Ps’ of in-store marketing (‘product’ availability, ‘price’, ‘promotion’ and ‘placement’) to favour healthiest over least healthiest food and drink options.^10^ Three studies conducted in two Australian states have demonstrated that targeted interventions to improve the nutrition of retail food environments can increase customer purchases of healthier items in sporting clubs and in recreation centres.^19^,^21^ However, the impact of these initiatives on overall sales revenue has been mixed. One randomised controlled trial of a multicomponent intervention targeting the availability and promotion of non-sugar-sweetened drinks and fruit and vegetable products in sporting clubs found no change in overall food and drink sales.^19^ Similarly, a pre-post intervention observational study of the introduction of a healthy food and drink policy in recreation centres found no change in food sales revenue but noted a 27% decrease in drink sales.^21^ Another observational study with a pre-post design also in recreation centres that only focused on drinks found that the sugar-sweetened beverage reduction initiative resulted in a 24% reduction in overall drink sales.^20^ These mixed results of shorter-term interventions highlight a need for longer-term trials in LG settings (both sporting clubs and recreation facilities), employing a rigorous study design and focused on both food and drinks.

Key barriers to implementation, maintenance and scaling-up healthy food provision identified through systems mapping include the lack of (1) skilled human resource time; (2) skill and knowledge-based capacity to drive change at the retail level; and (3) incentives for change.^22^ An evidence-based implementation framework to facilitate large-scale change was co-designed with project partners, involving 15 government and non-government organisations, to create the multicomponent ‘Promoting CHANGE’ support package (the intervention), to be tested in a 3-year type 2 effectiveness-implementation hybrid cluster randomised controlled trial (henceforth ‘trial’). Promoting CHANGE stands for ‘Promoting Community Health And Nutrition, and Government Engagement’.

The following protocol aims to describe the Promoting CHANGE trial which aims to investigate the implementation and effectiveness of the intervention on the healthiness of the food environment and sales in LG-owned and influenced sports and recreation facilities. We hypothesise that compared with Control facilities, facilities implementing Promoting CHANGE will lead to a reduction in (1) the percentage of the least healthiest food and drink products available estimated from products on display (implementation) and (2) the percentage of the least healthiest food and drink products purchased per week (effectiveness).

## Methods and analysis

### Study design

A type 2 effectiveness-implementation hybrid^23^ trial will run for 3 years beginning August 2023. Participant recruitment was completed before trial start, and data collection is ongoing (year 2 of 3 complete), with evaluation to occur mid 2026–2027. There are no restrictions on the implementation of other interventions or initiatives during the trial period, both in the intervention and control groups. The RE-AIM framework^24^ will be used to assess the reach, effectiveness, cost-effectiveness, adoption, degree of implementation and maintenance (sustainment) of Promoting CHANGE. Cost-effectiveness of Promoting CHANGE will be assessed by a within-study trial and modelling long-term intervention effects. The project logic model is attached as online supplemental appendix I. This trial protocol is reported in line with the SPIRIT statement^25^ and the checklist can be found in online supplemental appendix II.

### Patient and public involvement

All project partners, including participating LGs, have been involved in the intervention and implementation framework design and dissemination.

### Study setting

LGs in Victoria, Australia typically own and/or manage sport and recreation facilities such as sporting stadiums, gyms and indoor and outdoor pools. The financial relationships between LGs and these facilities vary by governance model; some may be internally run or directly operated by LGs, or alternatively leased to external providers under contractual or management agreements. In cases where facilities are externally managed, lease and service agreements may include financial incentives, performance requirements or funding arrangements that influence operational decisions. Consequently, changes in funding structures, contractual incentives or LG policy mandates could plausibly affect facility-level outcomes, although the extent of this influence would depend on the specific governance and contractual arrangements in place.

Many of these facilities have food outlets including cafes, kiosks and vending machines to which the voluntary Victorian Government ‘Healthy Choices’ guidelines^26^ for sports and recreation centres^27^ (hereafter ‘the Guidelines’) apply. The Guidelines classify food and drinks as ‘GREEN’ (best choices), ‘AMBER’ (choose carefully) or ‘RED’ (limit). Full compliance requires at least 50% of food and drink items available (displayed) in stores to be ‘GREEN’ and less than 20% to be ‘RED’.^27^ The Guidelines include requirements for the product availability, placement, pricing and promotion of food and drinks. These Guidelines are poorly implemented to date in sport and recreation settings.^28^

### Eligibility criteria

To be eligible, each LG was asked to recruit a minimum of two non-seasonal sports and recreation facilities that operate at least 10 months annually, each of which must include at least one food outlet. In addition, the facility managers were required to agree in principle to several conditions: (1) confirmation that they had not already fully implemented healthy in-store changes in line with the Guidelines^26^; (2) willingness to make food retail environment changes if assigned to the Intervention group; and (3) commitment to providing itemised sales data. If assigned to the Intervention group, LG-level representatives had to agree to fund at least 1 day per week of project officer time (with the remainder of their salary to be funded via the grant), along with manager supervision in-kind, over the trial period.

### Recruitment

Eight LGs, including 44 eligible non-seasonal facilities (three to nine per LG), were recruited for participation in the trial via an Expression of Interest process distributed to all 79 Victorian LGs with our partner networks between March and September 2021. LGs had the option to recruit additional facilities such as seasonal sporting clubs or non-seasonal libraries, arts centres and community centres to participate in the trial; however, these facilities would not be included in the evaluation of Promoting CHANGE. Recruitment occurred prior to a successful application for a federal government Partnership Project Grant from the Australian National Health and Medical Research Council (NHMRC).

### Intervention

**Figure 1:**
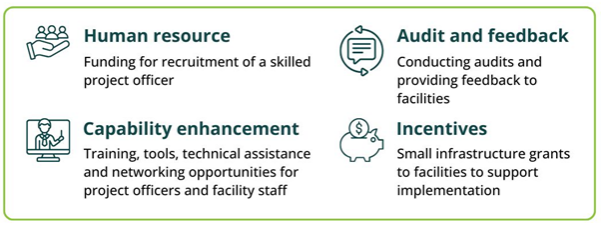
Promoting CHANGE intervention support package delivered over the 3-year trial period.

**Figure 2:**
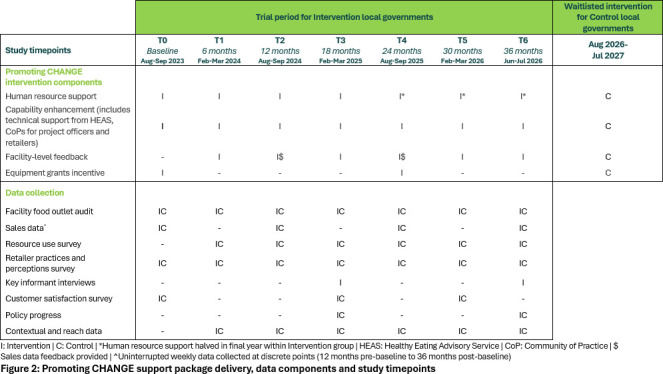
Promoting CHANGE support package delivery, data components and study timepoints.

A 3-year intervention was designed to support LG facilities towards compliance with the Guidelines. Components of the intervention were informed by a theoretical analysis of implementation determinants based on the Consolidated Framework for Implementation Research constructs,^29^ researchers’ previous experience and pilot studies^30^ and best-practice recommendations for multicomponent interventions from the Expert Recommendations for Implementing Change.^31^ Figure 1 shows the Promoting CHANGE support package components, figure 2 outlines when each of the components will be delivered during the trial and each component is described in detail in online supplemental appendix III within a Tidier table.^32^

#### Human resource support

The key component of the Promoting CHANGE support package involves salary support for skilled LG project officers trained in health promotion and/or facility management to deliver tailored, localised facilitation strategies.^33^ Project officer salary support was budgeted as half a day per week per LG for administrative tasks, evaluation, policy development, training and other capacity-building exercises, along with an additional half a day per facility for delivering tailored support to the sports and recreation facility food outlet. Examples of healthy choices implementation support include choosing specific new products based on supplier availability in the LG area and food preparation facilities within the outlet; marketing strategies tailored to customer base, for example, school carnivals for outdoor pools, demography of patrons. Of this, LGs agreed to fund 1 day worth of salary support and all staff on-costs; the NHMRC grant will fund the remainder of staff time. During the third year of the trial, grant-supplied salary support will be reduced by half as the project moves towards a long-term maintenance phase as indicated in figure 2.

LG project officers will collaborate with facility staff and management to advance implementation of the Guidelines. This includes creating or modifying recipes, identifying healthier product options and suppliers, training facility staff in improving the healthiness of the food outlet, creating communication and promotional materials and conducting six monthly in-store audits and collecting sales data. Decisions regarding which aspects of the Guidelines to prioritise will be made jointly by LGs and facilities with guidance from the Australian National Nutrition Foundation’s Healthy Eating Advisory Service (HEAS). LGs will be encouraged to draft policies aimed at embedding long-term changes, such as lease agreements and mandating compliance with the Guidelines.

#### Capability enhancement

The support package encompasses training sessions tailored for LG project officers and other relevant LG staff, delivered by HEAS with support from the research team and project partners, grounded in best practice principles for Community of Practice.^34^ These interactive training sessions will cover a spectrum of topics including cross-sectoral planning, engagement, policy development, health promotion, business skills and in-store marketing. The tools and technical assistance offered by HEAS include guidance on product classification using HEAS’ online FoodChecker classification tool,^35^ support for healthy food retail implementation planning and troubleshooting through a Microsoft Teams forum plus periodic telephone check-ins and provision of curated implementation and evaluation tools.

#### Feedback based on food outlet audit and sales data

Feedback will be provided by the research team for the LG project officers to present to intervention facilities. Facilities will receive six monthly verbal and written visual feedback based on food outlet audits including the healthiness of food and drinks on display, as well as customer satisfaction outcomes during the trial (at T0, T3). LGs will also receive feedback annually on the percentage of ‘GREEN’, ‘AMBER’ or ‘RED’ food and drinks sold each week based on units sold and weekly sales revenue. See figure 2 for when feedback delivery will occur for progress monitoring.

#### Incentives

Each facility will have access to $A1000 as small equipment grants to make enhancements to their food outlet to encourage healthier customer purchases during the 3-year trial.

### Control group

Facilities within LGs assigned to the Control group will continue with their current practice during the trial period, given the nature of the randomised controlled trial. Following the conclusion of the trial, Control group LGs will be offered a condensed 1-year long high-intensity human resource support package including project officer recruitment, equivalent financial incentives distributed over 1 year, skills and knowledge enhancement opportunities at project officer and facility level, auditing and feedback, along with equipment grant incentives, as benefit for participating in the trial.

### Outcome measures

For the purposes of this study, primary outcomes were selected based on the key policy focus of the Guidelines for sports and recreation settings, where full compliance requires at least 50% of food and drink items available in stores to be ‘GREEN’, and less than 20% to be ‘RED’.^27^

#### Primary outcomes

Implementation: At each facility, percentage of least healthiest ‘RED’ food and drink products available will be estimated from the products on display at the time of the audit. Effectiveness: At each facility, percentage of least healthiest ‘RED’ food and drink product volume (kg or L) purchased per week will be calculated using weekly sales data.

#### Secondary outcomes

*Secondary* measures are reported in online supplemental appendix IV (Section 1).

### Sample size considerations

Power calculations for sample size were performed in PASS (V.16). They were based on the number of LGs recruited and the number of non-seasonal facilities that agreed to provide display and point-of-sale data used to evaluate the sales outcomes. This included 21 non-seasonal facilities in four LGs in the Intervention group and 14 facilities in four LGs in the Control group (mean n=5 and n=4 sites per LG, respectively). *Primary implementation outcome*: The trial will have 80% power to detect a mean difference between groups of ≥18.8% in the percentage of ‘RED’ drinks displayed at the end of the 3-year trial (intraclass correlation coefficient 0.05, cluster size variation coefficient 0.37, SD=17.8, α=0.05, 2-sided test).^16 30^ This effect size is similar to that observed in the Water in Sport project—an uncontrolled pre-post natural experiment conducted in sports and recreation settings in Victoria, Australia (−18.9% at 18 months).^16 30^ *Primary effectiveness outcome*: With 156 weeks of sales data, the trial will have 80% power to detect a difference of ≥9% between groups in the percentage of ‘RED’ drink weekly drink sales at the end of the 3 year trial, using a generalised estimating equation (GEE) model assuming linear trends^36^ (first-order autoregressive, AR(1) correlation structure, ρ=0.73, SD=13.7). This effect size is smaller than in Water in Sport (−11% at 2 years).^30^ As of December 2024, due to the drop-out of one recruited LG and the withdrawal of some individual facilities in other LGs, the trial involves three LGs in the Intervention group, with a total of 16 non-seasonal facilities, and four LGs in the Control group, with a total of 8 non-seasonal facilities.

### Randomisation and blinding

Eight LGs were paired based on location (regional/urban), the socioeconomic index for areas,^37^ and the number of engaged facilities. At the end of September 2021, a statistician independent of the project produced the restricted random sequence to allocate LGs to Intervention or Control within pairs. The nature of the intervention precludes concealing LG allocation, as participants were informed of their grouping by the project lead. Data collectors, who are employees of the participating LGs, will not be blinded to intervention allocation. The study statistician will be blinded during analysis. After randomisation, one LG dropped out prior to the start of the trial in October 2022, citing altered LG capacity to participate.

### Data collection

Data collection will be conducted by project officers in Intervention LGs and nominated data collectors in Control LGs, who will receive annual training by the research team and HEAS to enhance the rigour of their data collection practices. The research team will also collect data independently at T0 and T6, to ensure objectivity of results for overall trial evaluation. This will also ensure that any final changes implemented within the intervention group, between T5 and trial end, will be captured.

#### Primary outcomes

##### Implementation

Facility photo audits will be conducted using a standardised protocol where photos of the whole outlet, along with detailed photos of each food and drinks section, promotional/advertising materials, menus and recipes, vending machines and all food and drinks stocked including nutrition panel will be captured every 6 months. For all pre-packaged products on display in each facility, photos will be used so that their healthiness can be ascertained. For products prepared onsite or where nutrition information is unknown, average nutrient content will be estimated using the Nutrition Panel Calculator by Food Standards Australia New Zealand^38^ or by matching each product to similar generic products within AUStralian Food and NUTrient Database (AUSNUT).^39^ All pre-packaged products, recipes for products prepared onsite and relevant nutrition information from photo audits will be entered into the FoodChecker web software^35^ which classifies each product as ‘GREEN’, ‘AMBER’ or ‘RED’ according to the Guidelines and determines the percentage of food and drinks on display in each of these three categories in each facility. FoodChecker assessments will be completed by project officers in the Intervention Group and by the research team for the Control group. At least 10% of the Intervention and Control group FoodChecker assessments will be cross-checked by the research team at T0 and T6.

##### Effectiveness

Weekly itemised sales data will be provided annually by each facility to track purchasing behaviours through electronic point-of-sale data. A detailed sales data extraction protocol will guide the procurement of sales data. Data collected will include date of sale, product name, cost and units sold. All available product names derived from sales codes will be matched with products displayed on site (where possible) and also classified for healthiness as ‘GREEN’, ‘AMBER’ or ‘RED’.

### Secondary outcomes

Data collection for secondary outcomes is described in online supplemental appendix IV (Section 3), including monitoring nutritional profile of available food and drinks, healthiness of the in-store food environment, resource use surveys to inform the cost-effectiveness of the support package and process evaluation measures including retailers’ practices and perceptions, relevant parties’ perceptions, customer satisfaction survey, LG policy progress, as well as contextual and reach data. Covariates for consideration are also outlined.

### Statistical methods

#### Primary outcomes

*Implementation*: Initially, the effect of Promoting CHANGE on each outcome will be assessed at the 3-year mark, using a GEE model^36^ to account for clustering within LGs, adjusted for facility characteristics. The GEE model framework will also be used to assess the effect of Promoting CHANGE on the six monthly outcomes over time.

*Effectiveness*: The effect of the intervention on the rate of change of weekly sales over the 3-year trial will be assessed using a GEE framework to account for autocorrelation in weekly trends over time. The model will include intervention, time and their interaction; facility as a fixed effect; and will adjust for weekly temperature and facility characteristics. Sensitivity analyses will also help handle any missing data. All analyses will be performed on an intention-to-treat basis, for all randomised participants for which there are available data.

#### Secondary outcomes

For analysis of secondary measures, see online supplemental appendix IV (Section 4).

## Discussion

### What is already known on this topic

Currently, only 2% of LGs in Australia have self-reported successfully implementing all the changes they desire to promote healthier food and drinks in their sports and recreation facilities.^28^ With over 500 LGs and approximately 6000 LG facilities with retail food outlets across Australia, the Promoting CHANGE initiative was developed to address barriers that prevent transformation of local food environments. Few LGs provide dedicated investment and support for implementation of healthy food retail strategies in sports settings, despite capacity building being a key element in many community change theories and frameworks.^31^ Additionally, Promoting CHANGE partner discussions and previous research^14 40^ suggest that more than 2 years of support is likely necessary to sustain momentum and maintain change, while evidence on the cost-effectiveness of healthy food retail initiatives remains limited.^41^ Evidence from supermarkets^42^ and remote Australian food stores^43^ indicates that auditing and providing feedback on in-store practices^44^ and commercial outcomes^45^ can encourage the adoption of best practice healthy food retail initiatives. To date, there have been no evaluations of the effectiveness of sales or food environment feedback mechanisms within a multicomponent support package in any community setting. Limited evidence of the value for money of retail-based health-promoting interventions^41^ suggests that future initiatives should include economic evaluations and the use of sales data as a proxy for consumption.

### What this study adds

This will be the first study to use a controlled design to test a multicomponent healthy food retail support package across a range of sports and recreational settings in LG settings. The findings from this trial will inform health promotion policy and practice across more than 500 Australian LGs, as well as community organisations including healthcare providers, schools and commercial retailers globally. This initiative will contribute to global efforts to enhance public health nutrition outcomes by responding to the WHO’s call for strengthened LG action in health promotion and food system reform.^3^

The Promoting CHANGE support package was co-designed with project partners using theoretically and empirically driven implementation approaches^24 29 31^ to address a significant partner-identified practice problem. It builds on prior research, including the 2-year VicHealth-initiated ‘Water in Sport’ project conducted across eight Victorian LGs which underscored the crucial role of project officers trained in public health in implementing healthy drink policies in sports settings.^30^ Comprehensive six monthly auditing and feedback on changes in the food retail environment, and tracking average weekly sales revenue, are key strengths of this study. By collecting outcomes expressed as being important by partners, such as their impact on sales, customer perspectives and value for money, study results can facilitate more effective resource allocation. These insights can provide a comprehensive understanding of which initiatives are most beneficial, ensuring that resources are directed toward areas that yield the greatest returns and align with food outlet objectives.

A rigorous cluster randomised and type 2 hybrid controlled trial design allows for the simultaneous examination of both the effectiveness of the support package and the processes involved in its implementation.^23^ This dual focus is particularly valuable to understand whether such an initiative works in real-world settings, and how and why it works or doesn’t work. Additionally, economic and process evaluations will provide a richer understanding of the factors influencing both the success and the implementation process. This could inform future scaling efforts and policy decisions in various retail settings, to influence global efforts to address key risk factors for non-communicable diseases.^46^,^48^

### Limitations of this study

LGs and facilities with a greater interest in nutrition may be more likely to participate in the study and implement changes. Moreover, staff turnover is a recurring issue at LG-run facilities. Turnover during the trial period may result in differences in data collection procedures and retail staff perceptions or practices and impact the accuracy of assessments of the in-store changes being made. The sustainment of the healthy changes implemented and provision of support at the LG level beyond the project’s life will depend on the LG priorities and capacity, the economic impact on facilities and the willingness of the retail staff to continue implementing healthy choices at their facilities. Care should be taken in the interpretation of this study’s results and the success of the intervention considering these limitations.

### Ethics and dissemination

Ethics approval was granted by the Deakin University Human Research Ethics Committee (reference number HEAG-H 92_2023). Organisational consent was secured from each LG for their participation by lead researcher (online supplemental appendix V), facility level consent was obtained by LG representatives on behalf of the research team (online supplemental appendix VI) and informed consent will be obtained by researchers and data collectors from individuals involved in any human data collection (including facility manager/staff during food environment audit (online supplemental appendix VII) and interviews (online supplemental appendix 8)), both written and verbal, depending on the nature of the project component for which data is being collected. For customer surveys, which will be anonymously collected, consent will be implied whereby participants will be informed that the completion/submission of the survey implies their consent. Any personal identifiers will remain confidential.

The study findings will be disseminated in open-access, peer-reviewed scientific journals. Plain language summaries will also be shared with participants.

### Data availability statement

All relevant data from this study will be made available on study completion on request. The datasets generated during and/or analysed during the current study are available from the corresponding author on reasonable request. Any personal identifiers will remain confidential during the data-sharing process, ensuring the privacy of participants.

## Supplementary material

10.1136/bmjopen-2025-109584 online supplemental file 1

10.1136/bmjopen-2025-109584 online supplemental file 2

10.1136/bmjopen-2025-109584 online supplemental file 3

10.1136/bmjopen-2025-109584 online supplemental file 4

10.1136/bmjopen-2025-109584 online supplemental file 5

10.1136/bmjopen-2025-109584 online supplemental file 6

10.1136/bmjopen-2025-109584 online supplemental file 7

10.1136/bmjopen-2025-109584 online supplemental file 8
