# Supplementary material for: Promoting CHANGE cluster randomised controlled trial to improve food outlet healthiness in Australian sport and recreation facilities: protocol
Source: BMJ Open. 2026 Mar 11;16(3):e109584. doi: 10.1136/bmjopen-2025-109584 (PMC12983731; doi:10.1136/bmjopen-2025-109584)
Supplement: online supplemental file 1 [file bmjopen-16-3-s001.pdf]

promoting CHANGE

Study aim: To assess the impact on Local Government (LG)-owned retail outlets, of a 3-year LG healthy food retail implementation support model (the Promoting CHANGE intervention) designed to improve the healthiness of the food environment, availability and sales of healthy food and drink, and if the intervention is effective and delivers good value for money

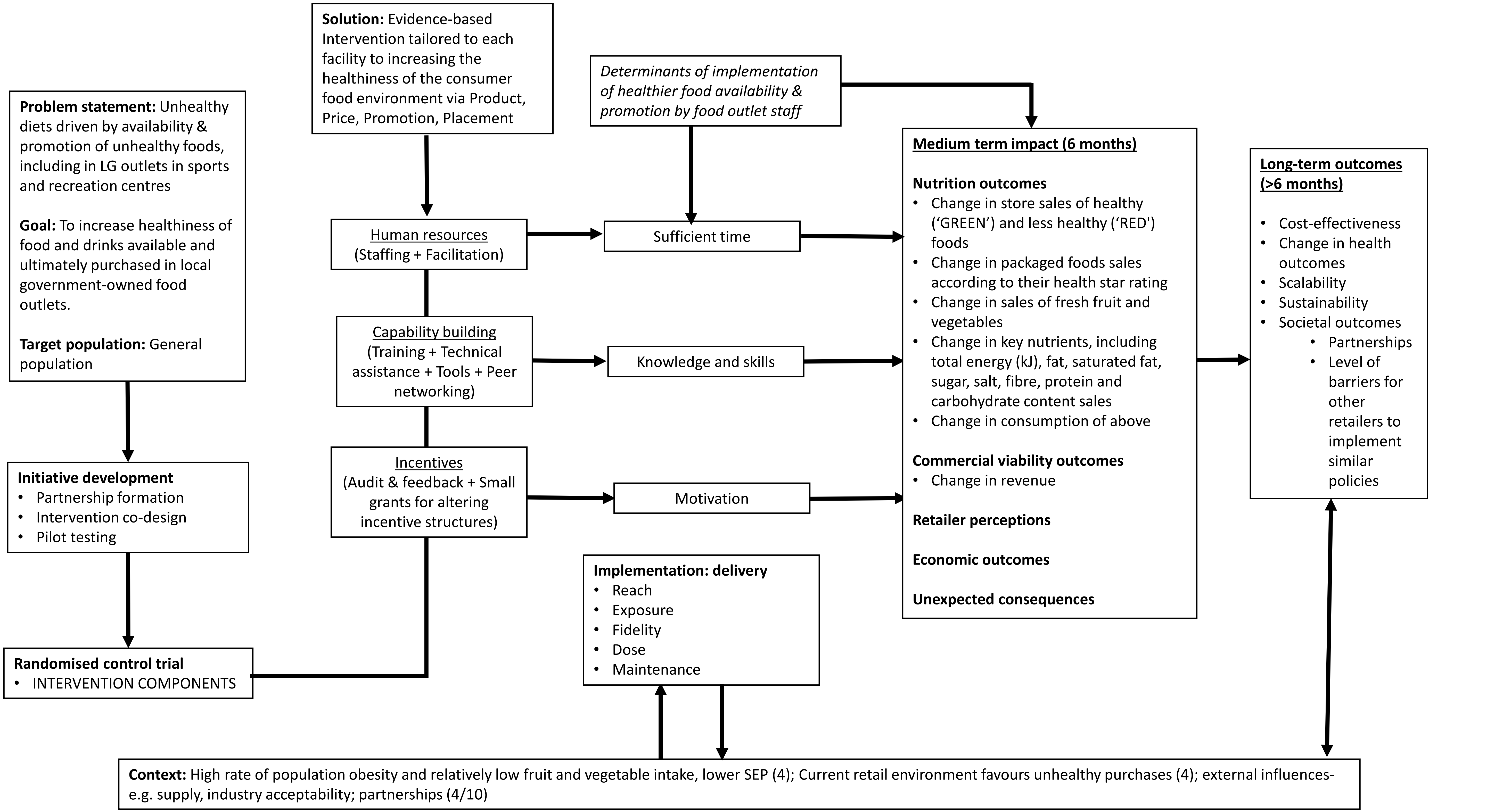

Deakin - Restricted to Staff
