# Supplementary material for: Promoting CHANGE cluster randomised controlled trial to improve food outlet healthiness in Australian sport and recreation facilities: protocol
Source: BMJ Open. 2026 Mar 11;16(3):e109584. doi: 10.1136/bmjopen-2025-109584 (PMC12983731; doi:10.1136/bmjopen-2025-109584)
Supplement: online supplemental file 3 [file bmjopen-16-3-s003.docx]

**Appendix II. Promoting CHANGE TIDieR table**

The Promoting CHANGE intervention constitutes the following support package components during the three-year trial period: 1) Providing human resource support through recruitment of a skilled local government (LG) project officer; 2) Offering training, tools, technical assistance, and peer networking opportunities through Community of Practice sessions to enhance project officer and facility staff capability; 3) Conducting audits and providing feedback throughout implementation to facilities regarding the healthiness of the food environment and the proportion of unhealthy food and beverages purchased; and 4) Offering small grants to facilities as incentives to further improvements on site for healthy choices implementation.

At the end of the trial period, the Control group will be offered a shortened one-year version of the Promoting CHANGE Intervention, with support to encourage sustainable changes.

Further details on **the Promoting CHANGE intervention components to be delivered** are outlined below:

| **Intervention component name** | **Details** | **Description** |
| --- | --- | --- |
| **Onboarding package** | **Who receives** | Control and Intervention LG representatives, intervention project officers, nominated data collectors in control groups |
|  | **Rationale** | To provide an overview of the project and the project resources available and expected timelines to meet project deliverables |
|  | **Materials** | Onboarding document with project and data collection resources linked, includes project purpose, governance, and timelines |
|  | **Intervention provider** | Research team |
|  | **Modes of delivery (including location)** | Email |
|  | **When and how much** | Beginning of intervention and for any new staff during trial |
|  | **Measurement of delivery including tailoring and modifications** | None |
| **Human resourcing** | **Who receives** | Intervention LGs for project officer recruitment |
|  | **Rationale** | Skilled LG project officers in the intervention LGs during the trial period (salary-supported) providing best-practice, tailored, localised facilitation strategies, plus data collection. |
|  | **Materials** | Financial support to LGs. LGs to provide funding for ~50% of the role cost, remainder provided by the grant |
|  | **Intervention provider** | LG-level recruitment prior to trial period |
|  | **Modes of delivery (including location)** | Online or in-person interviews for new recruits; internal LG-level recruitment where applicable |
|  | **When and how much** | Half a day per week as a base per LG (time for administration, evaluation, policy development, training, other capacity-building exercises) PLUS half a day per facility for delivering tailored support (total funding 2-5 days per week per LG depending on number of facilities).  In the third year (maintenance period), support will be halved. |
|  | **Measurement of delivery including tailoring and modifications** | Full Time Equivalent measured through health economics evaluation |
| **Human resource support** | **Who receives** | Control LGs for data collector recruitment |
|  | **Rationale** | Nominated data collectors in control LGs during the trial period to collect data |
|  | **Materials** | Financial support provided by grant |
|  | **Intervention provider** | LG |
|  | **Modes of delivery (including location)** | Online or in-person interviews for new recruits; internal LG-level recruitment where applicable |
|  | **When and how much** | For data collection, 4 hours per 6 months per facility (approx. 90mins onsite, plus travel time, training and offsite data entry) |
|  | **Measurement of delivery including tailoring and modifications** | Health economics evaluation of resources used to implement intervention components, through logbook completion by project officers |
| **Training tools** | **Who receives** | Intervention project officers and nominated data collectors in control groups |
|  | **Rationale** | Training to conduct food environment audits, sales data procurement; additional FoodChecker training to assess menus and products for Intervention project officers only |
|  | **Materials** | Online training, emails containing data collection components with project and data collection resources linked; additional evaluation tools (e.g. web-based customer surveys). |
|  | **Intervention provider** | Research team and HEAS |
|  | **Modes of delivery (including location)** | In-person and recorded training sessions  organised by Research team and HEAS; Access to resources via dedicated Microsoft Teams group |
|  | **When and how much** | Beginning of intervention; twice for Intervention project officers for FoodChecker assessment training; ongoing as required |
|  | **Measurement of delivery including tailoring and modifications** | Number of attendees at training sessions |
| **Technical support for capability enhancement** | **Who receives** | Intervention project officers |
|  | **Rationale** | Tools and technical assistance to overcome knowledge gaps by covering a spectrum of topics including cross-sectoral planning, stakeholder engagement, policy development, health promotion, business skills, and in-store marketing, to ensure sustained capacity building. |
|  | **Materials** | Technical assistance to assist with change implementation, and curated implementation and evaluation tools (e.g. policy evaluation templates) |
|  | **Intervention provider** | Research team and HEAS |
|  | **Modes of delivery (including location)** | Online/in-person support meetings and periodic telephone check-ins; Access to resources via dedicated Microsoft Teams group |
|  | **When and how much** | Via 6-monthly telephone check-ins on progress; as needed via Microsoft Teams group. |
|  | **Measurement of delivery including tailoring and modifications** | Captured via process evaluation interviews with project officers |
| **Peer networking through CoP sessions for project officers** | **Who receives** | Intervention project officers |
|  | **Rationale** | To encourage/ motivate peer discussions of change implementation progress, share ideas and challenges, means to seek support and identify training opportunities for capability enhancement |
|  | **Materials** | N/A |
|  | **Intervention provider** | HEAS led, Research team supported |
|  | **Modes of delivery (including location)** | Online and in-person sessions |
|  | **When and how much** | Quarterly project officer CoPs alternating between 1-hour informal online mode and 4 hours in-person formal mode |
|  | **Measurement of delivery including tailoring and modifications** | Number of sessions held and number of attendees |
| **Peer networking through CoP sessions for facility staff members** | **Who receives** | Intervention facility staff members |
|  | **Rationale** | To encourage/ motivate peer discussions of change implementation progress, share ideas and challenges, means to seek support and identify training opportunities for capability enhancement |
|  | **Materials** | N/A |
|  | **Intervention provider** | Project officer led, HEAS and Research team supported |
|  | **Modes of delivery (including location)** | Hybrid in-person and online allowing for maximum attendance |
|  | **When and how much** | Six-monthly facility CoP 90-minute hybrid mode (facility staff members at their respective LGs gather in-person and then cross-fertilise discussions occur online with other LGs) |
|  | **Measurement of delivery including tailoring and modifications** | Number of sessions held and number of attendees |
| **In-store food environment and sales outcomes feedback** | **Who receives** | Project Officers who then disseminate to participating facilities during their intervention period |
|  | **Rationale** | Feedback will allow sites to make iterative changes to improve Promoting CHANGE effectiveness and reduce unintended financial consequences and will facilitate applications to local accreditation programs to gain recognition and further support. |
|  | **Materials** | Verbal and written feedback to assist with development of facility specific targets and action plan towards implementation of the Guidelines. Feedback includes in-store food environment healthiness scores, % of GREEN/AMBER/RED food and drinks available as displayed, photos for visual record of changes, % of GREEN/AMBER/RED food and drinks sold, weekly mean sales revenue |
|  | **Intervention provider** | Research team |
|  | **Modes of delivery (including location)** | Online between research team and project officer using PowerPoint slide deck; usually in-person between project officer and facility staff/manager |
|  | **When and how much** | Every six months, through 1 hour feedback meetings between research team and project officer; project officer then meets with facility staff/manager on site to discuss |
|  | **Measurement of delivery including tailoring and modifications** | Number of feedback sessions delivered |
| **Small equipment grants** | **Who receives** | Participating facilities during intervention year |
|  | **Rationale** | To incentivise change and support altering food environments to make them more health-enabling |
|  | **Materials** | Equipment grants application templates, purchase invoices |
|  | **Intervention provider** | Research team |
|  | **Modes of delivery (including location)** | As monies via LG |
|  | **When and how much** | $1000; in two instalments of $500 each, post baseline data collection, and at the 2-year point of the project |
|  | **Measurement of delivery including tailoring and modifications** | Date of equipment purchased for change implementation, record of purchase amount and item; Some sites have accessed the entire grant amount post-baseline due to the cost of equipment purchased |
| **Newsletter** | **Who receives** | Project partners, LGs, and relevant stakeholders, interested parties |
|  | **Rationale** | Updates on timelines, staffing and intervention progress to maintain engagement |
|  | **Materials** | Emailed newsletter |
|  | **Intervention provider** | Research team |
|  | **Modes of delivery (including location)** | Email update |
|  | **When and how much** | Every three months |
|  | **Measurement of delivery including tailoring and modifications** | Number of subscribers, opens, clicks |

Based on TIDieR checklist: Hoffmann T, Glasziou P, Boutron I, Milne R, Perera R, Moher D, Altman D, Barbour V, Macdonald H, Johnston M, Lamb S, Dixon-Woods M, McCulloch P, Wyatt J, Chan A, Michie S. Better reporting of interventions: template for intervention description and replication (TIDieR) checklist and guide. BMJ. 2014;348:g1687.

**Abbreviations**

CoP: Community of Practice

HEAS: Healthy Eating Advisory Service

LG: Local Government
