# Supplementary material for: Promoting CHANGE cluster randomised controlled trial to improve food outlet healthiness in Australian sport and recreation facilities: protocol
Source: BMJ Open. 2026 Mar 11;16(3):e109584. doi: 10.1136/bmjopen-2025-109584 (PMC12983731; doi:10.1136/bmjopen-2025-109584)
Supplement: online supplemental file 4 [file bmjopen-16-3-s004.docx]

Appendix IV: Promoting CHANGE secondary outcome measures, data collection, and analysis

# Secondary outcome measures

## Adoption, implementation and maintenance

Adoption will be determined as any change to the healthiness of in-store product availability, promotion, placement and/or price (the ‘4Ps’). Implementation and maintenance will also capture the extent and duration of any change to the 4Ps.

## Effectiveness

Effectiveness measures at each facility will include weekly percentage of ‘GREEN’ and ‘AMBER’ food and drink volume (kg or L) purchased, percentage of ‘GREEN’, ‘AMBER’ and ‘RED’, food and drinks unit purchased, total food and drink revenue (AU$), and nutritional content of products purchased (including mean Health Star Rating (HSR) and nutrient content per 100g/ml (energy (kJ), protein (g), fat (g), saturated fat (g), carbohydrate (g), total sugar (g), added sugar (g), dietary fibre (g), sodium (mg)). HSR is a voluntary front-of-pack nutrient profiling and labelling system in Australia and New Zealand [1]. Products receive a HSR from 0.5 stars (least healthy) to 5 (healthiest).

## Cost-effectiveness

Cost-effectiveness will be evaluated by analysing the short-term (over the trial duration) cost and resource use to deliver the intervention per percentage change in the availability and sale of ‘RED’ items, alongside modelled long-term cost-effectiveness based on improved healthiness of purchases.

## Process factors

Process factors will be examined including community, facility staff, and LG perspectives on the impact of implementation support over time, mechanisms of impact for each of the Promoting CHANGE support strategies, and relevant parties’ perceptions of the factors necessary to enhance implementation and sustainment of Promoting CHANGE. Customer surveys at baseline, mid- and end-trial will examine acceptance and purchasing behaviour, providing valuable insights into consumer preferences and responses to healthier options. Strength of LG policies that support healthy, equitable, and sustainable food systems will also be assessed through process evaluation.

## Reach

Reach will be measured through customer attendance data, including sales transactions and units purchased, as available.

# Covariates

For each facility we will collect food outlet type (café, kiosk, vending machine); location (urban/rural); level of disadvantage (Socio-Economic Indexes for Areas); and weekly mean daily temperatures. Since the response to the intervention might differ by facility type, we will consider that covariate in our exploratory analysis.

# Data collection

## Adoption, implementation and maintenance

Baseline (T0) and six-monthly food outlet audits until T6 will be used to measure adoption (any facility change from T1 to T6 compared to T0), implementation (extent of change at T0-T3) and maintenance (extent of change from T3 to T6) using the ‘Café Scout’ tool. Café Scout was adapted from the ‘Store Scout app’, originally designed for remote grocery settings [2] and administered via REDCap online survey tool [3, 4] (hosted at Deakin University). Café Scout assesses the ‘4Ps of marketing’ (product availability, placement, price, and promotion) in cafés, kiosks, and vending machines. The tool evaluates the healthiness of the food outlet and product offerings, asking up to 278 Yes/No questions, across eight product categories (16-48 questions per product category): ‘Pre-packaged drinks’, ‘Drinks prepared onsite’, ‘Non-perishable snacks’, ‘Fresh, refrigerated and frozen snacks’, ‘Cold meals’, ‘Hot food’, ‘Vending: drinks’ and ‘Vending: snacks’. Upon completion of the survey, an overall score out of 100 (0= least healthy, 100= healthiest possible score) will be calculated for each intervention facility as well as individual scores for each ‘4P of marketing’ and product category.

Contextual data will be captured at T1-T6 via REDCap surveys on facility characteristics such as: 1) any actions taken on specific food outlet changes planned during previous six months; 2) changes to menu; 3) new initiatives or specials; 4) special events; and 5) outlet closures or opening outside usual hours. Data will be used to confirm all relevant healthy changes have been captured within on-site audits and assist with interpretation of sales data outliers.

Audits will be completed by LG-employed project officers in the Intervention group and LG-employed data collectors in the Control group. To ensure calibration and validation, audits at baseline and a final additional time point at the end of the trial (T6) will be conducted by at least two independent tool users at the same outlets: one LG-level auditor and one research team member.

## Effectiveness

Point-of-sale sales data will be used to calculate the secondary outcomes that measure effectiveness. The energy content and HSR of items sold will be calculated using the facility photo audits that were collected to monitor the nutritional profile of available food and drinks. Photographs of front-of-pack and back-of-pack labels of packaged products available in each facility will be used to extract information on: product name, brand, serving size, and nutrient content per 100g/ml (energy (kJ), protein (g), fat (g), saturated fat (g), carbohydrate (g), total sugar (g), added sugar (g), dietary fibre (g), sodium (mg)). Additional product information will be collected including the product’s name as per sales data codes (accounting for name changes over time), and its HSR. If the HSR is not available on packaging, it will be calculated using the latest version of the HSR calculator [5]. For freshly prepared foods, the HSR will be determined using the calculator based on the estimated nutrition information of the closest generic product from the AUStralian Food and NUTrient Database (AUSNUT) [6].

## Cost-effectiveness

Cost and resources used to implement all intervention components will be collected. Data collection tools include administrative data (e.g., invoices for small equipment grants), log books (implemented in Microsoft Excel) completed by LG project officers detailing the time and costs associated with the Promoting CHANGE activities, continuous logbooks completed by research staff capturing the time required to complete the data analysis for the feedback component of the Promoting CHANGE intervention, and telephone interviews with Healthy Eating Advisory Service support staff (starting at T1 until T6). A REDCap survey, appended to Café Scout, will collect data on the time and resources used by facilities to implement any changes resulting from the intervention (from T1 to T6). Interviews within the process evaluation (detailed below) will assess whether any health promotion activities were undertaken in Control LG sports and recreation facilities and the associated resource.

Additionally, the customer survey will help estimate the proportion of facility users whose purchasing behaviour changes in response to the Promoting CHANGE intervention. This includes consumers who substitute unhealthy products with healthier options within the facility, as well as those who purchase similar unhealthy products elsewhere. These data will be used to estimate the population that benefits from the intervention.

## Process evaluation

### Food retailer practices and perceptions survey

Store practices and food outlet staff perspectives will be collected within the Café Scout tool every six months during the trial (starting at T0 until T6). Facility managers or staff in charge will be asked to assess the extent to which their store supports a healthy food environment, rated on a 5-point Likert scale (from 1=not at all, 5=a lot), and outlet policies related to product, placement, pricing, and promotional offerings. Some questions are of Yes/No types (some have 'Not sure' or 'N/A' response options), and others are sliding scale questions to measure how important certain healthy food outlet practices are from the store manager's perspective.

### Key informant interviews

To assess relevant parties’ perceptions, approximately 20 semi-structured interviews will be conducted to evaluate how the outcomes of the Promoting CHANGE intervention are influenced by: 1) the implementation processes of each strategy; 2) mechanisms of impact; and 3) the LG context, such as socioeconomic status. These interviews will target a purposive sample of food outlet staff, LG project officers and managers, and others involved in supporting implementation at the mid- and end-point of the trial from the Intervention group. Strategy adaptations used to address implementation barriers, and relevant parties’ perceptions of Promoting CHANGE costs and benefits, its acceptability, and the support and infrastructure needed for long-term maintenance, sustainment, and scale-up will be investigated at T3 and T6.

### **Customer satisfaction survey**

Customer feedback will be gathered using a short, anonymous survey in both Intervention and Control facilities (at T0, T3 and T5), with an option for Control facilities to continue collection of customer feedback during their intervention period after the trial. Survey questions include: *1) How happy are you with the food and drink offerings at this food outlet today? [Option to choose between Very Happy, Happy, Neutral, Unhappy, Very Unhappy] and 2) Tell us more! What would you like to see more or less of in our food and drink offerings?*. QR codes linking to a Qualtrics survey will be placed in prominent areas such as checkouts within each food outlet for up to four weeks at each timepoint, and participants could go into a draw to win a gift voucher worth AU$50.

### Policy progress

The effectiveness of LG policies in supporting healthy, equitable, and sustainable food systems will be evaluated using an abbreviated version of the Local Food-EPI+ tool [7]. Local Food-EPI+ uses publicly available and internal policy documents to benchmark LG performance against best practice indicators in various domains. Within Promoting CHANGE, the relevant policy domains identified include ‘Leadership’, ‘Funding and resources’, ‘Monitoring and intelligence’, ‘Food promotion’, ‘Food provision and retail in LG facilities and public spaces’, and ‘Supermarkets and food sources in the community’. Researchers will collate internal and external-facing council policy documents from Intervention and Control LGs at T3 and T6.

## Reach

Reach data will be collected at each six-monthly data collection round except baseline (at T1-T6). To assess Reach in both Intervention and Control facilities, sales data will also be used to measure customer attendance by analysing the number of transactions where available and the total units of products purchased.

## Covariates

Outlet type (café, kiosk, vending machine) will be captured via REDCap surveys. Publicly available data will capture local government and facility characteristics, including location (urban/rural) using Accessibility/Remoteness Index of Australia (ARIA+) [8], area level of disadvantage using Socio-Economic Indexes for Areas (SEIFA) [9], and weekly mean daily temperatures from the Australian Bureau of Meteorology [10].

# Analysis of secondary outcomes

## Adoption, implementation and maintenance

To measure adoption, we will perform a simple count of the number of facilities that improved their food environment between T0 and T3 as evidenced by increases to facility Café Scout scores, and/or decreases in the raw percentage of ‘RED’ food and drinks on display and/or increases in the raw percentage of ‘GREEN’ food and drinks on display. A generalised estimating equations (GEE) model will be fitted to evaluate healthiness of the in-store environment (implementation and maintenance outcomes) for Intervention compared to Control facilities at all collected time points. This model will account for the clustering of facilities within LGs and adjust for the covariates listed in Section 2.

## Effectiveness

The same analysis approach used to evaluate the primary effectiveness outcomes will be used to evaluate the secondary effectiveness outcomes.

## Cost-effectiveness

A within-trial and modelled cost-effectiveness evaluation will be undertaken. For the within-trial analysis, the incremental cost of Intervention implementation compared to Controls per percentage reduction in the availability and sales of ‘RED’ items over the three-year trial period will be presented. The long-term cost-effectiveness of the Promoting CHANGE intervention will be assessed using the ACE-Obesity Policy model [11] adapted to the Victorian state population. The ACE-Obesity Policy model is a multiple cohort Markov lifetable model. It has been used to assess the cost-effectiveness of various obesity prevention interventions in the Australian setting [11]. The percentage reduction in total energy sold will be modelled to estimate changes in energy intake among the impacted population, and the resulting changes in body mass index (BMI), using the same methods described in previous published economic evaluations [12]. The modelling involves the following steps:

- Estimating the population impacted: A component of the customer survey (described under section 3.4.3) will inform the proportion of individuals attending the facility who change their purchasing behaviour as a result of the intervention, specifically those who substituted unhealthy options with healthier alternatives. The survey also identifies the proportion of consumers who substituted with similar unhealthy products external to the facility. These proportions will inform the size of the population who benefit from the Intervention.
- Translating energy sold to energy consumed: The change in energy sold will be converted into changes in daily energy intake, informed by purchasing frequency data obtained from the survey and supplemented by literature-based estimates. Certain assumptions will be applied depending on results of literature search.
- Converting energy intake to change in body weight: Changes in energy intake will be translated into changes in body weight using the validated estimate that a sustained change of 100 kilojoules per day corresponds to a 1 kg change in body weight over 3 years [13].
- Estimating changes in BMI: Weight changes will be converted to BMI changes using average Australian height and weight data stratified by gender and age groups.

The ACE-Obesity Policy model will predict the impact of the BMI changes on the epidemiology of nine obesity related diseases (type 2 diabetes, ischaemic heart disease, hypertensive heart disease, stroke, osteoarthritis of the hip and knee and four cancers: breast, colorectal, kidney and endometrial). The change in epidemiology of diseases results in changes in the quantity and quality of life of Australians (quantified as health-adjusted life years (HALYs)) and healthcare cost savings.

The analyses will be undertaken using a limited societal perspective and outputs will include intervention costs, HALYs gained, healthcare cost-savings, and incremental cost-effectiveness ratios. Changes in facility revenue/profits will not be included in the analysis under the assumption that change in sales will be redistributed to unimpacted sectors and products [14] with minimal impact on overall economy. For the scaled-up modelling where the intervention will be implemented across all Victorian LGAs, additional cost components and efficiencies related to program management and coordination will be estimated using data from both the trial and relevant literature.

Unintended consequences identified in the process evaluation will be incorporated where possible into the economic analysis and described qualitatively if these are unable to be quantified and valued.

## Process evaluation

### Food retailer practices and perceptions survey

GEE modelling framework will be used to compare the managers responses between Intervention and Control facilities over time (at T0-T6) using linear regression to analyse the Likert scales with 5 points.

### Key informant interviews

Thematic analysis of the interview data will occur deductively, guided by the Consolidate Framework for Implementation Research (CFIR) constructs [15], RE-AIM framework [16] and the Intervention Scalability Assessment Tool [17].

### **Customer satisfaction survey**

GEE linear regression models will be used to examine whether customer satisfaction changes over time (baseline, midpoint, endpoint of trial) between Intervention and Control facilities.

### Policy progress

A qualitive assessment of strength of LG policy will be assessed by descriptively comparing each LG policy domain and overall performance against Local Food-EPI+ best practice indicators.

## Reach

A GEE modelling approach will be used to assess reach and differences between Intervention and Control facilities over time (at T0-T6).

# References

1. Department of Health. Health Star Rating System Canberra, Australia: Australian Department of Health; 2019 [cited 2024 28 November]. Available from: <http://healthstarrating.gov.au/>.

2. McMahon EJ, Jaenke R, Brimblecombe J. A mobile app to rapidly appraise the in-store food environment: reliability, utility, and construct validity study. JMIR mHealth uHealth. 2020;8(7):e16971.

3. Harris PA, Taylor R, Minor BL, Elliott V, Fernandez M, O'Neal L, et al. The REDCap consortium: Building an international community of software platform partners. J Biomed Inform. 2019;95:103208. Epub 2019/05/13. doi: 10.1016/j.jbi.2019.103208. PubMed PMID: 31078660; PubMed Central PMCID: PMCPMC7254481.

4. Harris PA, Taylor R, Thielke R, Payne J, Gonzalez N, Conde JG. Research electronic data capture (REDCap)—A metadata-driven methodology and workflow process for providing translational research informatics support. J Biomed Inf. 2009;42(2):377-81. doi: <https://doi.org/10.1016/j.jbi.2008.08.010>.

5. Department of Health. Health Star Rating calculator Canberra, Australia: Australian Department of Health; 2014 [cited 2024 28 November]. Available from: <http://www.healthstarrating.gov.au/internet/healthstarrating/publishing.nsf/Content/excel-calculator>.

6. Food Standards Australia New Zealand. AUSNUT 2011–13–Australian food composition database. Canberra: FSANZ; 2014.

7. Ruffini O, Relf C, Mann D, Blake MR, Carrad A, Reeve B, et al. Development of the Local Food Systems Policy Index (Local Food-EPI+) tool and assessment process to benchmark the implementation of local government policies for creating healthy, equitable and environmentally sustainable food systems. Public Health Nutr. 2024;27(1):e191.

8. Australian Bureau of Statistics. Remoteness Areas: ABS; Jul2021-Jun2026 [cited 2024 19 December]. Available from: <https://www.abs.gov.au/statistics/standards/australian-statistical-geography-standard-asgs-edition-3/jul2021-jun2026/remoteness-structure/remoteness-areas>.

9. Australian Bureau of Statisitics. Socio-Economic Indexes for Areas (SEIFA), Australia [Internet] Canberra: ABS; 2021 [cited 2024 11 August]. Available from: <https://www.abs.gov.au/statistics/people/people-and-communities/socio-economic-indexes-areas-seifa-australia/latest-release>.

10. Australian Bureau of Meteorology. Climate Data Online Canberra, Australia: Bureau of Meteorology; 2021 [cited 2024 28 November]. Available from: <http://www.bom.gov.au/climate/data-services/station-data.shtml>.

11. Ananthapavan J, Sacks G, Brown V, Moodie M, Nguyen P, Veerman L, et al. Priority-setting for obesity prevention—The Assessing Cost-Effectiveness of obesity prevention policies in Australia (ACE-Obesity Policy) study. PLoS One. 2020;15(6):e0234804.

12. Lal A, Mantilla-Herrera AM, Veerman L, Backholer K, Sacks G, Moodie M, et al. Modelled health benefits of a sugar-sweetened beverage tax across different socioeconomic groups in Australia: A cost-effectiveness and equity analysis. PLoS Med. 2017;14(6):e1002326.

13. Hall KD, Sacks G, Chandramohan D, Chow CC, Wang YC, Gortmaker SL, Swinburn BA. Quantification of the effect of energy imbalance on bodyweight. Lancet. 2011;378(9793):826-37. doi: 10.1016/S0140-6736(11)60812-X.

14. Siggins Miller. Consultancy services to inform the development of a Post Implementation Review of the tobacco plain packaging measure. Regulatory Burden Measurement & Analysis of Costs and Benefits. Post Implementation Review (PIR) [Internet]. , 2016 [21 January 2026]. Available from: <https://oia.pmc.gov.au/published-impact-analyses-and-reports/tobacco-plain-packaging>.

15. Damschroder LJ, Aron DC, Keith RE, Kirsh SR, Alexander JA, Lowery JC. Fostering implementation of health services research findings into practice: a consolidated framework for advancing implementation science. Implement Sci. 2009;4(1):50.

16. Glasgow RE, Vogt TM, Boles SM. Evaluating the public health impact of health promotion interventions: the RE-AIM framework. Am J Public Health. 1999;89(9):1322-7.

17. Lee K, Milat A, Grunseit A, Conte K, Wolfenden L, Bauman A. The Intervention Scalability Assessment Tool: a pilot study assessing five interventions for scalability. Public Health Res Pract. 2020;30(2):e3022011.
