# Supplementary material for: Promoting CHANGE cluster randomised controlled trial to improve food outlet healthiness in Australian sport and recreation facilities: protocol
Source: BMJ Open. 2026 Mar 11;16(3):e109584. doi: 10.1136/bmjopen-2025-109584 (PMC12983731; doi:10.1136/bmjopen-2025-109584)
Supplement: online supplemental file 5 [file bmjopen-16-3-s005.docx]

| **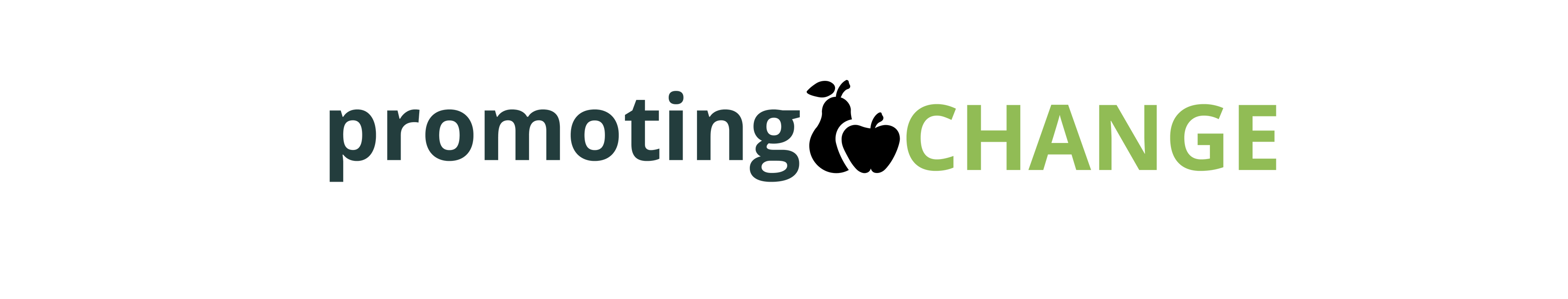PLAIN LANGUAGE STATEMENT AND CONSENT FORM** | 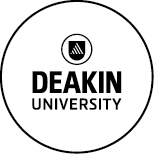 |
| --- | --- |

**TO: LOCAL GOVERNMENTS AND EXTERNAL MANAGEMENT COMPANIES**

**Plain Language Statement**

**Date: 25 July 2023**

Full Project Title: Promoting CHANGE randomised controlled trial

**Principal Researcher: Dr Miranda Blake**

**Associate Researcher(s):** **A/Prof Adrian Cameron, Dr Jaithri Ananthapavan, Dr Helena Romaniuk, Prof Liliana Orellana, Dr Neha Lalchandani, Bettina Backman, Dr Pam Nguyen**

**What is this Plain Language Statement and Consent Form about?**

This Plain Language Statement contains detailed information about the “Promoting CHANGE randomised controlled trial” research project. Its purpose is to explain to you as openly and clearly as possible the procedures involved in this project so that you can make a fully informed decision about whether you are going to participate. Please read this Plain Language Statement carefully. You may also wish to discuss the project with a relative or friend. Once you understand what the project is about and if you agree to take part in it, you can continue with the study. You should save or print off a copy of the Plain Language Statement to keep as a record.

**What is the purpose of this research project?**

Promoting CHANGE is a joint initiative led by Deakin University, in partnership with City of Greater Bendigo, City of Greater Geelong, City of Greater Shepparton, City of Merri-bek, City of Monash, City of Yarra, Maroondah City Council, Menzies School of Health Research, Monash University, Municipal Association of Victoria, Nillumbik Shire Council, Nutrition Australia (Victorian Division), Sport & Recreation Victoria, The University of Newcastle, and the Victorian Department of Health. Promoting CHANGE is supported by a National Health and Medical Research Council (NHMRC) Partnership Project Grant (GNT2015440).

Promoting CHANGE aims to encourage community healthy eating by providing support to local governments to improve the healthiness of foods for sale in local government facilities. To investigate if this support can improve the healthiness of foods and drinks available, the healthiness of foods purchased, and the effects on revenue for facilities, the Promoting CHANGE randomised controlled trial will run from mid-2023 to mid-2027.

The project covered by this participant information sheet and consent form will collect data on the current healthiness of food and drinks available and promoted in the facility (called the ‘food environment’), healthiness of food and drinks sold to customers, and outlet revenue for food and drinks. This data will be used to compare differences in outcomes between the intervention and control groups, over time.

**What data is being collected?**

From the facilities that participate in the Promoting CHANGE project, the following data will be collected:

- Food environment data includes: Food product availability, pricing, promotion (marketing) and placement. This will be assessed by researchers using tools such as:
- Store Scout Kiosk tool, which collects data on the food and drink environment (e.g., products, placement in display cabinet, price and promotion) and surveys store managers to obtain an overview of the in-store food environment and store practices. Facility managers or staff in charge will be asked for their responses to questions regarding the perceived importance of providing a healthy food environment to customers and their perceptions to which this is currently happening in their store.
- Photographs of food displays (note that no humans will be included in photographs).
- The store menu and list of any other food and drinks offered, plus their ingredients, and recipes if prepared onsite.
- Itemised weekly sales data for each product available, e.g., number of items sold and price per item (where available)
- Information on costs and staff resources to implement the intervention from facility staff/managers as part of the health economics evaluation survey.
- Interviews with intervention retail outlet staff, LG project officers and managers, and other stakeholders at the mid- and end-points of the RCT will assess how the implementation outcomes of the Promoting CHANGE framework.
- Anonymous customer satisfaction survey responses facilitated by QR codes installed at the facility.
- Local-government level policy documents.

**What does participating involve?**

For local governments and external management companies, participating involves providing contact details for facilities previously identified as having agreed to participate in Promoting CHANGE. Facility food environment data and food and drink sales data will be collected by researchers and/or recruited project officers at intervention facilities or local employees at control facilities.

**What are the possible benefits and risks of participating?**

Local government-owned food service outlets are considered key players in contributing to healthy environments for their communities. By participating in this project, you will help us to evaluate the effectiveness of the Promoting CHANGE project to support facilities to promote community healthy eating. It is not expected that you or your organisation will be exposed to any physical or commercial risk, or psychological discomfort by participating in this project. Sales data will be treated in strictest commercial confidence, as below. Researchers will adhere to all relevant COVID-19 restrictions.

**What will happen to the data provided?**

All identifiable information on yourself and your organisation, including sales data, will remain confidential, meaning that identifiable information (including your name, job position or organisation) will not be accessible by anyone outside of the Research Team. Results and external documents will ensure the data is non-identifiable. Your local government will be identified in the methods and acknowledgments of any external communication. Facilities will only be identifiable to that facility and governing local government, unless there is prior agreement to share identifiable information more broadly. De-identified responses may be shared with other researchers for research purposes only.

All electronica data will be stored in a secure drive, and paper-based data in local filing cabinets, within the School of Health and Social Development at Deakin University. Data will be stored securely for five years following scientific publication, after which all data will be destroyed.

**What will happen to the research findings?**

Following the completion of the trial and analysis of results, lay summaries will be emailed to the managers of all participating facilities, and to key local government contacts. Individual participants will not be provided with specific feedback. Findings for each facility will also be shared with the local government which owns that facility.

**Is participation in this research voluntary?**

Participation in any research project is voluntary. If your organisation does not wish to take part in this study, it is not obliged to. The decision to participate will not affect your or your organisation’s relationship with Deakin University or the research team. Once you have commenced the study you can withdraw at any time prior to the analysis of the data. If you would like to withdraw, please contact the researchers using the contact details below advising that you no longer wish to take part. Upon this request any data you have provided will not be used. Participating in this study does not mean you have to participate in later research if you do not want to.

**What ethical guidelines are being followed in this research?**

This project will be carried out according to the National Statement on Ethical Conduct in Human Research (2007) updated in 2018, produced by the National Health and Medical Research Council of Australia. This statement has been developed to protect the interests of people who agree to participate in human research studies. Deakin University’s Human Research Ethics Committee has approved this research project.

**Who should I contact if I have any complaints about the research?**

If you have any complaints about any aspect of the project, the way it is being conducted or any questions about your rights as a research participant, then you may contact:

The Human Research Ethics Office, Deakin University, 221 Burwood Highway, Burwood Victoria 3125, Telephone: 9251 7129, [research-ethics@deakin.edu.au](mailto:research-ethics@deakin.edu.au)

Please quote project number HEAG-H 92_2023.

**Who should I contact for further information, queries, or any problems?**

Promoting CHANGE research team:

Global Centre for Preventive Health and Nutrition (GLOBE)

Deakin University

221 Burwood Highway, Burwood, VIC 3125

Telephone: +61 3 9246 8487 (Dr Miranda Blake) or +61 3 9244 5438 (Neha Lalchandani)

Email (to both Miranda and Neha): [promotingchange@deakin.edu.au](mailto:promotingchange@deakin.edu.au)


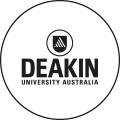
**
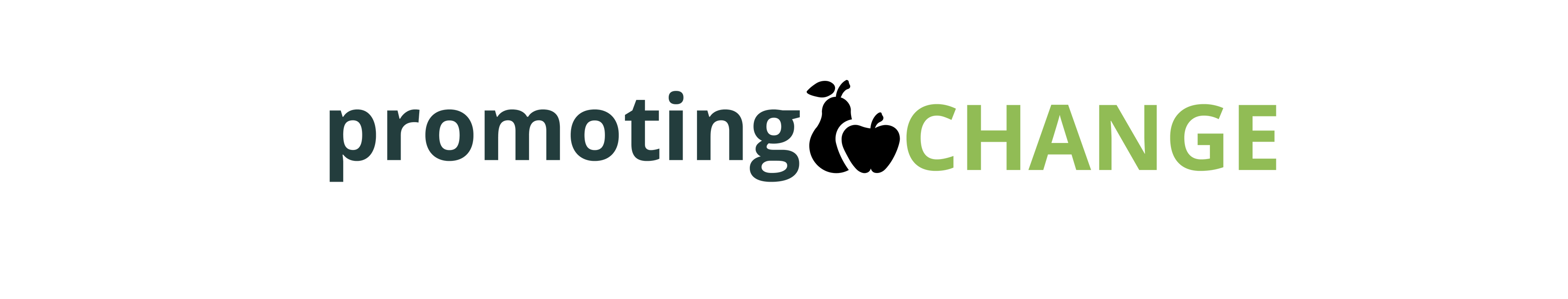
TO: LOCAL GOVERNMENTS AND EXTERNAL MANAGEMENT COMPANIES**

**Organisational Consent Form**

*(To be used by organisational employee with the relevant authority providing consent for staff members/volunteers to be involved in research)*

**Date: 25 July 2023**

**Full Project Title:** **Promoting CHANGE randomised controlled trial**

**Reference Number: HEAG-H 92_2023**

I have read, and I understand the attached Organisational Plain Language Statement*.*

I give my permission for my organisation to participate in this project according to the conditions in the Plain Language Statement and agree to support the project as described in the Organisational Plain Language Statement, including (where I have authority to do so): the provision of facility contact details, agreement for facilities to participate, and provision of food and drink sales data.

I have been given a copy of the Organisational Plain Language Statement and Consent Form to keep.

The researchers have agreed not to reveal the participants’ identities and personal details if information about this project is published or presented in any public form.

I have the authority to provide consent on behalf of this organization to participate in this research project.

Participant Name (printed) ………………………………………………………

Signature ……………………………………………………… Date…………………………

There are two options for returning consent via email to Dr Miranda Blake and Neha Lalchandani at: [promotingchange@deakin.edu.au](mailto:promotingchange@deakin.edu.au), either:

1. Signing of consent forms and returning via email to Dr Miranda Blake and Neha Lalchandani at: [promotingchange@deakin.edu.au](mailto:promotingchange@deakin.edu.au), OR

2. Copying and pasting the consent form text into an email and sending to the research team at [promotingchange@deakin.edu.au](mailto:promotingchange@deakin.edu.au).
