# Supplementary material for: Promoting CHANGE cluster randomised controlled trial to improve food outlet healthiness in Australian sport and recreation facilities: protocol
Source: BMJ Open. 2026 Mar 11;16(3):e109584. doi: 10.1136/bmjopen-2025-109584 (PMC12983731; doi:10.1136/bmjopen-2025-109584)
Supplement: online supplemental file 7 [file bmjopen-16-3-s007.docx]

| **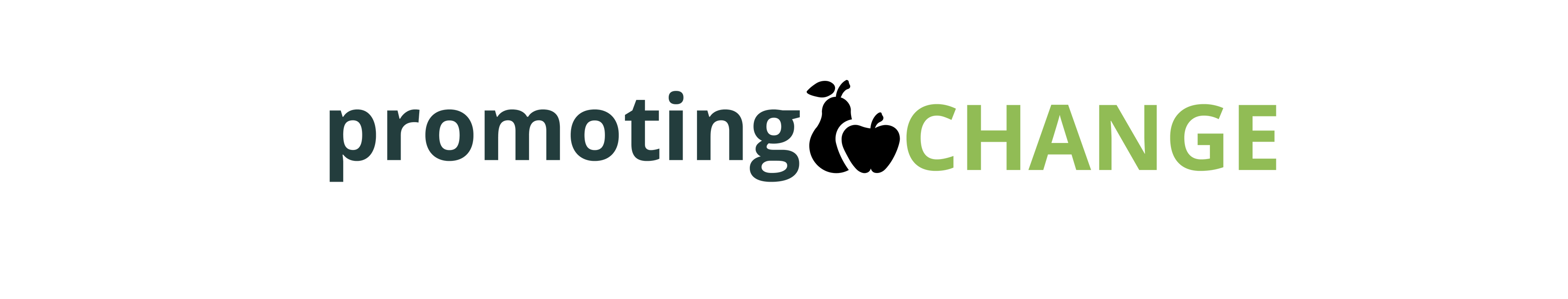PLAIN LANGUAGE STATEMENT AND CONSENT FORM** | 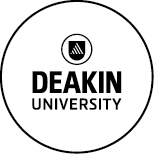 |
| --- | --- |

**TO: FACILITY STAFF REGARDING FACILITY VISITS AND SURVEYS**

**Plain Language Statement**

**Date: 25 July 2023**

**Full Project Title: Promoting CHANGE randomised controlled trial**

**Principal Researcher: Dr Miranda Blake**

**Associate Researcher(s):** **A/Prof Adrian Cameron, Dr Jaithri Ananthapavan, Dr Helena Romaniuk, Prof Liliana Orellana, Dr Neha Lalchandani, Bettina Backman, Dr Pam Nguyen**

**What is this Plain Language Statement and Consent Form about?**

You have been identified as knowing important information relating to the food offerings at your facility which may assist in understanding the healthiness of foods available and sold at your facility. This Plain Language Statement contains detailed information about the “Promoting CHANGE randomised controlled trial” research project. Its purpose is to explain to you as openly and clearly as possible the procedures involved in this project so that you can make a fully informed decision about whether you are going to participate. Please read this Plain Language Statement carefully. You may also wish to discuss the project with a relative or friend. Once you understand what the project is about and if you agree to take part in it, you can continue with the study. You should save or print off a copy of the Plain Language Statement to keep as a record.

**What is the purpose of this research project?**

Promoting CHANGE is a joint initiative led by Deakin University, in partnership with City of Greater Bendigo, City of Greater Geelong, City of Greater Shepparton, City of Merri-bek, City of Monash, City of Yarra, Maroondah City Council, Menzies School of Health Research, Monash University, Municipal Association of Victoria, Nillumbik Shire Council, Nutrition Australia (Victorian Division), Sport & Recreation Victoria, The University of Newcastle, and the Victorian Department of Health. Promoting CHANGE is supported by a National Health and Medical Research Council (NHMRC) Partnership Project Grant (GNT2015440).

Promoting CHANGE aims to encourage community healthy eating by providing support to local governments to improve the healthiness of foods for sale in local government facilities. To investigate if this support can improve the healthiness of foods and drinks available, the healthiness of foods purchased, and the effects on revenue for facilities, the Promoting CHANGE randomised controlled trial will run from mid-2023 to mid-2027. Your facility has agreed in-principle to participate in the Promoting CHANGE project via its governing local government.

The project covered by this participant information sheet and consent form will collect data on the importance of providing a healthy food environment to customers and whether the project is enabling that.

**What data is being collected?**

As facility manager or staff in charge, you will be asked for your opinions regarding the importance of providing a healthy food environment to customers your perceptions as to what extent this is currently happening in your store. You may also be invited to participate in a survey to understand your time/resource use and cost effectiveness of the intervention. These questions will be collected by university researchers or local government employees every six months during the project.

**What does participating involve?**

For relevant facility staff this will include 30 mins - 1 hour to answer the survey questions, every 6 months during the project (mid-2023 to mid-2026).

**What are the possible benefits and risks of participating?**

Local government-owned food service outlets are considered key players in contributing to healthy environments for their communities. By participating in this project, you will help us assess the impact of the Promoting CHANGE Intervention on the healthiness of the food environment, its adoption and acceptability of changes. It is not expected that you or your organisation will be exposed to any physical or commercial risk, or psychological discomfort by participating in this project. You will not receive any personal reimbursement for participating. Researchers will adhere to all relevant COVID-19 restrictions.

**What will happen to the data provided?**

All identifiable information on yourself and your organisation will remain confidential, meaning that identifiable information (including your name, job position or organisation) will not be accessible by anyone outside of the Research Team. Results and external documents will ensure the data is non-identifiable. Your local government will be identified in the methods and acknowledgments of any external communication. Facilities will only be identifiable to that facility and governing local government, unless there is prior agreement to share identifiable information more broadly. De-identified responses may be shared with other researchers for research purposes only.

All electronic data will be stored in a secure drive, and paper-based data in local filing cabinets, within the School of Health and Social Development at Deakin University. Data will be stored securely for five years following scientific publication, after which all data will be destroyed.

**What will happen to the research findings?**

Following the completion of the trial and analysis of results, lay summaries will be emailed to the managers of all participating facilities, and to key local government contacts. Individual participants will not be provided with specific feedback.

**Is participation in this research voluntary?**

Participation in any research project is voluntary. If you do not wish to take part in this study, you are not obliged to. The decision to participate will not affect your relationship with Deakin University or the research team. Once you have commenced the study you can withdraw at any time prior to the analysis of the data. If you would like to withdraw, please contact the researchers using the contact details below advising that you no longer wish to take part. Upon this request any data you have provided will not be used. Participating in this study does not mean you have to participate in later research if you do not want to.

**What ethical guidelines are being followed in this research?**

This project will be carried out according to the National Statement on Ethical Conduct in Human Research (2007) updated in 2018, produced by the National Health and Medical Research Council of Australia. This statement has been developed to protect the interests of people who agree to participate in human research studies. Deakin University’s Human Research Ethics Committee has approved this research project.

**Who should I contact if I have any complaints about the research?**

If you have any complaints about any aspect of the project, the way it is being conducted or any questions about your rights as a research participant, then you may contact:

The Human Research Ethics Office, Deakin University, 221 Burwood Highway, Burwood Victoria 3125, Telephone: 9251 7129, [research-ethics@deakin.edu.au](mailto:research-ethics@deakin.edu.au)

Please quote project number HEAG-H 92_2023.

**Who should I contact for further information, queries, or any problems?**

Promoting CHANGE research team:

Global Centre for Preventive Health and Nutrition (GLOBE)

Deakin University

221 Burwood Highway, Burwood, VIC 3125

Telephone: +61 3 9246 8487 (Dr Miranda Blake) or +61 3 9244 5438 (Neha Lalchandani)

Email (to both Miranda and Neha): promotingchange@deakin.edu.a


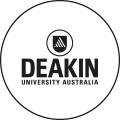
**
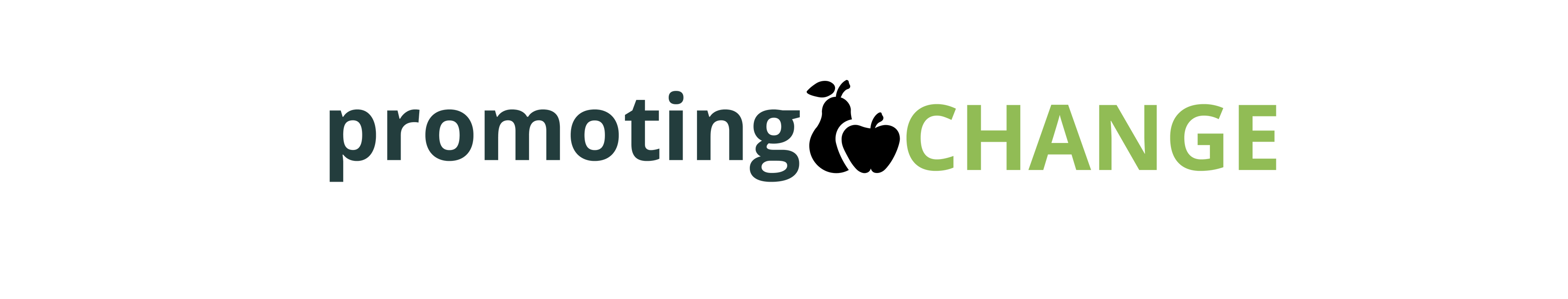
TO: FACILITY STAFF**

**Consent Form**

**Date: 25 July 2023**

**Full Project Title: Promoting CHANGE randomised controlled trial**

**Reference Number: HEAG-H 92_2023**

I have read, and I understand the attached Plain Language Statement*.*

I give my permission to participate in this project according to the conditions in the Plain Language Statement.

I have been given a copy of the Plain Language Statement and Consent Form to keep.

The researcher has agreed not to reveal the participants’ identities and personal details if information about this project is published or presented in any public form.

Participant Name (printed) ………………………………………………………

Signature ……………………………………………………… Date…………………………

There are three options for returning consent:

1. Signing of consent forms and returning via email to Dr Miranda Blake and Neha Lalchandani at: [promotingchange@deakin.edu.au](mailto:promotingchange@deakin.edu.au), OR

2. Copying and pasting the consent form text into an email and sending to the research team at [promotingchange@deakin.edu.au](mailto:promotingchange@deakin.edu.au), OR

3. Electronic consent by signing on an electronic tablet on the day of data collection.
