# Supplementary material for: Promoting CHANGE cluster randomised controlled trial to improve food outlet healthiness in Australian sport and recreation facilities: protocol
Source: BMJ Open. 2026 Mar 11;16(3):e109584. doi: 10.1136/bmjopen-2025-109584 (PMC12983731; doi:10.1136/bmjopen-2025-109584)
Supplement: online supplemental file 8 [file bmjopen-16-3-s008.docx]

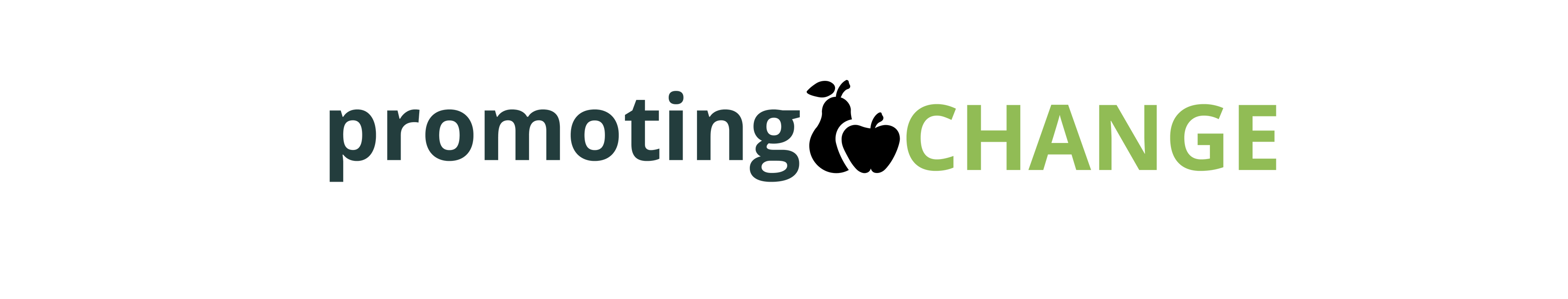

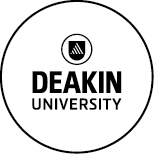


TO: INTERVIEW PARTICIPANT

**Plain Language Statement**

Date: 17 March 2025

Full Project Title: Promoting CHANGE randomised controlled trial

**Principal Researcher: Dr Miranda Blake**

**Associate Researcher(s):** **Prof Adrian Cameron, A/Prof Jaithri Ananthapavan, Dr Helena Romaniuk, Prof Liliana Orellana, Dr Neha Lalchandani, Bettina Backman, Dr Pam Nguyen, Ms Anne Barrow, Ms Nitong Jian, Dr Victoria Brown**

**What is this Plain Language Statement and Consent Form about?**

You have been identified as someone whose perceptions are valuable to understand the acceptability of the Promoting CHANGE project, its impacts, and what is needed for its long-term maintenance and scale-up. This Plain Language Statement contains detailed information about the “Promoting CHANGE randomised controlled trial” research project. Its purpose is to explain to you as openly and clearly as possible the procedures involved in this project so that you can make a fully informed decision about whether you are going to participate. Please read this Plain Language Statement carefully. You may also wish to discuss the project with a relative or friend. Once you understand what the project is about and if you agree to take part in it, you can continue with the study. You should save or print off a copy of the Plain Language Statement to keep as a record.

**What is the purpose of this research project?**

Promoting CHANGE is a partnership between the Global Centre for Preventive Health and Nutrition (GLOBE) at Deakin University, the Victorian Department of Health, Municipal Association of Victoria, National Nutrition Foundation, Sport and Recreation Victoria, the Cities of Greater Bendigo, Greater Geelong, Greater Shepparton, Maroondah, Monash, Merri-bek, Nillumbik and Yarra.

Promoting CHANGE aims to encourage community healthy eating by providing support to local governments to improve the healthiness of foods for sale in local government facilities. To investigate if this support can improve the healthiness of foods and drinks available, the healthiness of foods purchased, and the effects on revenue for facilities, we plan to run the Promoting CHANGE randomised control trial in 2023-27.

The current project covered by this participant information sheet and consent form will collect data on the acceptability of the Promoting CHANGE intervention, its impacts, and perceptions on enhancing its sustainability and scalability over time.

**What data is being collected?**

Through the interviews, conducted at two time points over the duration of the project, we aim to find out what those involved in Promoting CHANGE implementation think about the Promoting CHANGE approach, how well the different project components work, and how the approach could be improved to be maintained over time.

**What does participating involve?**

You are invited to participate in an audio and video-recorded interview which will be conducted in person or online via Zoom/MS Teams. During the interview you will be asked about your thoughts and experiences with healthy food changes in food manufacturing, distribution or retail, which may include the ‘Promoting CHANGE’ intervention. Participating will involve a total 45-60 minutes of your time to answer interview questions and may include a 15-minute survey prior to the interview. Following your interview, you will be emailed a copy of the interview transcript and will have two weeks to reply with any comments or changes you would like to provide. We may also invite you to participate in a second interview the following year. You are not obliged the complete the follow-up interview if you change your mind.

**What are the possible benefits and risks of participating?**

Local government-owned food service outlets are considered key players in contributing to healthy environments for their communities. By participating in this project, you will help us assess the uptake and acceptability of the Promoting CHANGE Intervention, its impacts and long-term sustainability and scalability. It is not expected that you or your organisation will be exposed to any physical or commercial risk, or psychological discomfort by participating in this project. You will receive a $50 Prezzee voucher (or similar) for participating for each interview, that can be used at a range of stores.

**What will happen to the data provided?**

All identifiable information on yourself and your organisation will remain confidential, meaning that identifiable information (including your name, job position or organisation) will not be accessible by anyone outside of the Research Team. Results and external documents will ensure the data is non-identifiable. Local governments will be identified in the methods and acknowledgments of any external communication. Facilities will only be identifiable to that facility and governing local government, unless there is prior agreement to share identifiable information more broadly.

All electronic data will be stored in a secure drive, and paper-based data in locked filing cabinets, within the School of Health and Social Development at Deakin University. Data will be stored securely for five years following scientific publication, after which all data will be destroyed. De-identified data may be used by other researchers for research purposes.

**What will happen to the research findings?**

Participants can contact the researchers to request a lay summary of de-identified interview findings this by emailing researchers at [promotingchange@deakin.edu.au](mailto:promotingchange@deakin.edu.au). These will be provided following project data collection completion and analysis.

**Is participation in this research voluntary?**

Participation in any research project is voluntary. If you do not wish to take part in this study, you are not obliged to. The decision to participate will not affect your relationship with Deakin University or the research team. Once you have commenced the study you can withdraw at any time prior to the analysis of the data. If you would like to withdraw, please contact the researchers using the contact details below advising that you no longer wish to take part. Upon this request any data you have provided will not be used. Participating in this study does not mean you have to participate in later research if you do not want to.

**What ethical guidelines are being followed in this research?**

This project will be carried out according to the National Statement on Ethical Conduct in Human Research (2023), produced by the National Health and Medical Research Council of Australia. This statement has been developed to protect the interests of people who agree to participate in human research studies. Deakin University’s Faculty of Health Human Ethics Advisory Group has approved this research project.

**Who should I contact if I have any complaints about the research?**

If you have any complaints about any aspect of the project, the way it is being conducted or any questions about your rights as a research participant, then you may contact:

The Human Research Ethics Office, Deakin University, 221 Burwood Highway, Burwood Victoria 3125, Telephone: 9251 7129, [research-ethics@deakin.edu.au](mailto:research-ethics@deakin.edu.au)

Please quote project number HEAG-H 92_2023.

**Who should I contact for further information, queries, or any problems?**

Promoting CHANGE research team:

Global Centre for Preventive Health and Nutrition (GLOBE)

Deakin University

221 Burwood Highway, Burwood, VIC 3125

Telephone: +61 3 9246 8487 (Dr Miranda Blake) or +61 3 9244 5438 (Dr Neha Lalchandani)

Email (to both Miranda and Neha): [promotingchange@deakin.edu.au](mailto:promotingchange@deakin.edu.au)


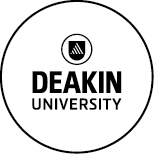
**
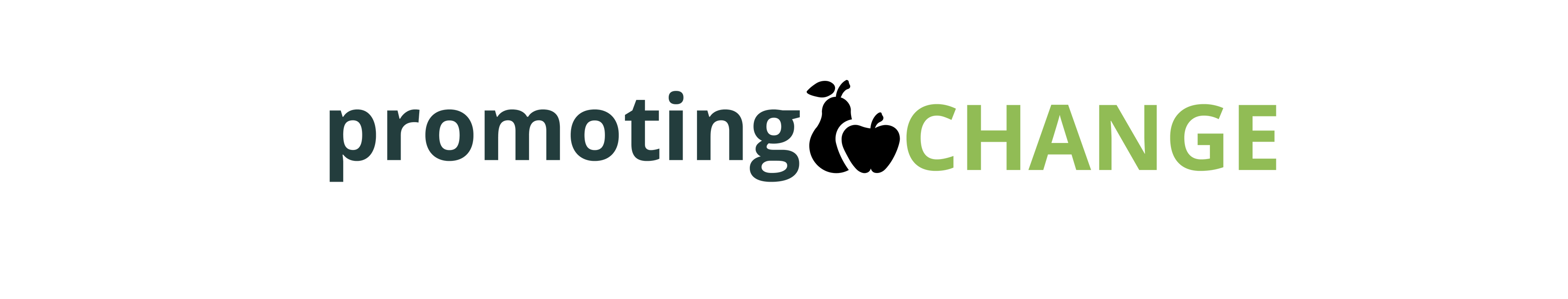
**

**TO: INTERVIEW PARTICIPANT**

**Consent Form**

Date: 17 March 2025

Full Project Title: Promoting CHANGE randomised controlled trial

**Reference Number: HEAG-H 92_2023**

I have read, and I understand the attached Plain Language Statement*.*

I give my permission to participate in this project according to the conditions in the Plain Language Statement.

I have been given a copy of the Plain Language Statement and Consent Form to keep.

The researcher has agreed not to reveal the participants’ identities and personal details if information about this project is published or presented in any public form.

Participant Name (printed) ………………………………………………………

Signature ……………………………………………………… Date…………………………

There are two options for returning consent:

1. Providing consent via the online form: <https://researchsurveys.deakin.edu.au/jfe/form/SV_9HJwkemsD6iBcOO>

2. Copying and pasting the consent form text into an email and sending to the research team, Dr Miranda Blake and Dr Neha Lalchandani at: [promotingchange@deakin.edu.au](mailto:promotingchange@deakin.edu.au)

**
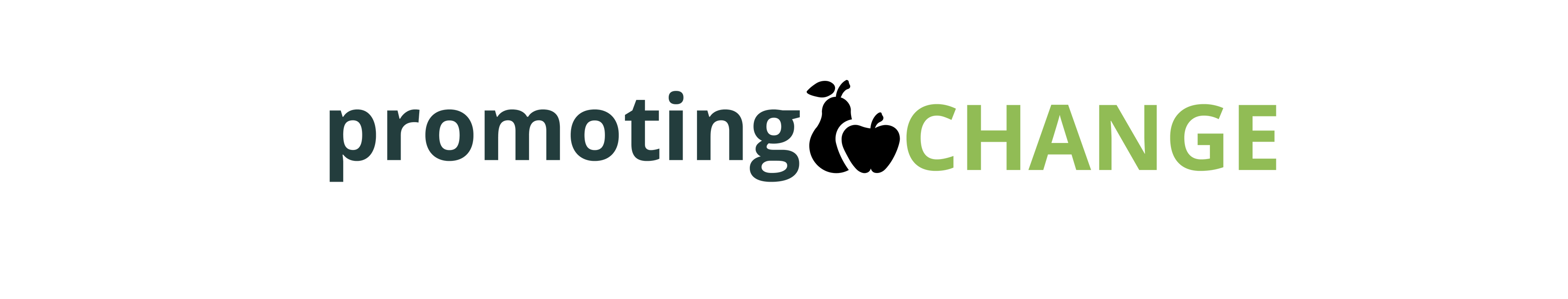
**
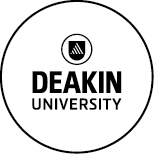


**TO: INTERVIEW PARTICIPANT**

**Withdrawal of Consent Form**

Date: 17 March 2025

Full Project Title: Promoting CHANGE randomised controlled trial

**Reference Number: HEAG-H 92_2023**

I hereby wish to WITHDRAW my consent to participate in the above research project and understand that such withdrawal WILL NOT jeopardise my relationship with Deakin University.

Participant Name (printed) ………………………………………………………

Signature ……………………………………………………… Date…………………………

**Please post or email this form to:**

Promoting CHANGE research team:

Global Centre for Preventive Health and Nutrition (GLOBE)

Deakin University

221 Burwood Highway, Burwood, VIC 3125

Telephone: +61 3 9246 8487 (Dr Miranda Blake) or +61 3 9244 5438 (Dr Neha Lalchandani)

Email (to both Miranda and Neha): [promotingchange@deakin.edu.au](mailto:promotingchange@deakin.edu.au)
