# Supplementary material for: Promoting CHANGE cluster randomised controlled trial to improve food outlet healthiness in Australian sport and recreation facilities: protocol
Source: BMJ Open. 2026 Mar 11;16(3):e109584. doi: 10.1136/bmjopen-2025-109584 (PMC12983731; doi:10.1136/bmjopen-2025-109584)
Supplement: Supplementary file 9 [file bmjopen-2025-109584.reviewer_comments.pdf]

PEER REVIEW HISTORY

BMJ Open publishes all reviews undertaken for accepted manuscripts. Reviewers are asked to complete a checklist review form and are provided with free text boxes to elaborate on their assessment. These free text comments are reproduced below.

ARTICLE DETAILS

Title (Provisional)

Promoting CHANGE cluster randomised controlled trial to improve food outlet healthiness in Australian sport and recreation facilities: Protocol

Authors

Lalchandani, Neha; Romaniuk, Helena; Cameron, Adrian; Orellana, Liliana; Ananthapavan, Jaithri; Peeters, A; Backman, Bettina; Adam, Megan; Allender, Steven; Nguyen, Phuong; Sacks, Gary; Brimblecombe, Julie Kay; McMahon, Emma; Blake, Miranda

---

VERSION 1 - REVIEW

**Reviewer**	**1**
**Name**	**Chan, Sze Ling**
**Affiliation**	**Singhealth, Health Services Research Centre**
**Date**	**22-Dec-2025**
**COI**	**None**

The problem is a worthy one to tackle, the rationale is strong and the study well-designed and described. I have a few comments and questions.

1. Line 175-176: What is the total number of LGs and facilities in the partner network? This provides information on adoption (which should be % of LGs willing to implement the intervention). The reasons for non-participation and characteristics of participating and non-participating LGs may be relevant for adoption in future scale up.

2. Explain more on how the intervention is tailored to each facility

3. Since LGs own the facilities, what is the financial relationship between them? Is it possible to tie the outcome to financial incentives/funding or mandate change?

4. Covariates – How about type of facility? The patrons at gyms and pools might are likely different from sporting stadiums, especially if they are there as spectators.

**Reviewer**	**2**
**Name**	**Healey, Andy**

**Affiliation**	**King's College London, IOPPN**
**Date**	**05-Jan-2026**
**COI**	**None**

Thank you for your manuscript.

I only have minor comments and suggestions for revision both concerning the planned economic evaluation (my area of expertise) of which details were supplied in the supplementary material:

It would be helpful to have more clarity on how the primary and secondary end points in your hybrid study will be translated into the clinical metrics that will be used to make long-term "HALY" and health system cost extrapolations using the obesity model you intend to use. For example, how will total energy sold and energy intake be estimated from your study data and then transformed into BMI for the modelling?

If the modelling will extrapolate impacts at the Victoria State population level, then presumably this means that you would need to model the costs of implementing the intervention at scale as as well (i.e. beyond the confines of the hybrid trial). Again, please clarify your intensions here.

Will impact of the intervention on overall (total) sales revenues from the food outlets as measured through your sales data be factored in to the economic evaluation? Again, please clarify.

In relation to the above point, it wasn't clear whether or to what extent the study would measure unintended consequences of the intervention that may be relevant to an economic assessment. For example, if a higher % of sales were for green category products but total sales revenue had fallen, this may be because some people had substituted towards other nearby food outlets selling more unhealthy items (fast food outlets or whatever).

VERSION 1 - AUTHOR RESPONSE

Reviewer 1 Comments	Author responses
The problem is a worthy one to tackle, the rationale is strong and the study well-designed and described. I have a few comments and questions.	Thank you kindly for your feedback and recognising our work.
1. Line 175-176: What is the total number of LGs and facilities in the partner network? This provides information on adoption (which should be % of LGs willing to implement the intervention). The reasons for non-participation and characteristics of participating and non-	Added lines 181-182 “distributed to all 79 Victorian local governments”.

participating LGs may be relevant for adoption in future scale up.	
2. Explain more on how the intervention is tailored to each facility	Added lines 208-212 "Examples of healthy choices implementation support include choosing specific new products based on supplier availability in the local government area and food preparation facilities within the outlet; marketing strategies tailored to customer base e.g., school carnivals for outdoor pools, demography of patrons."
3. Since LGs own the facilities, what is the financial relationship between them? Is it possible to tie the outcome to financial incentives/funding or mandate change?	Added lines 150-158 "The financial relationships between LGs and these facilities vary by governance model; some may be internally run or directly operated by LGs, or alternatively leased to external providers under contractual or management agreements. In cases where facilities are externally managed, lease and service agreements may include financial incentives, performance requirements, or funding arrangements that influence operational decisions. Consequently, changes in funding structures, contractual incentives, or LG policy mandates could plausibly affect facility-level outcomes, although the extent of this influence would depend on the specific governance and contractual arrangements in place."
4. Covariates – How about type of facility? The patrons at gyms and pools might are likely different from sporting stadiums, especially if they are there as spectators.	We agree that the response to the intervention might differ by facility type. Hence this will be considered as a covariate in our exploratory analysis. Added lines 39-40 within the secondary outcomes supplementary file (Appendix IV).

**Reviewer 2 Comments**	**Author responses**
Thank you for your manuscript.<br>I only have minor comments and suggestions for revision both concerning the planned economic evaluation (my area of expertise) of which details were supplied in the supplementary material:	We thank the reviewer for the insightful comments. We have revised the manuscript to clarify the modelling method in Appendix IV (lines 178-219), reflecting the explanation provided in our response below.
It would be helpful to have more clarity on how the primary and secondary end points in your hybrid study will be translated into the clinical metrics that will be used to make long-term "HALY" and health system cost extrapolations using the obesity model you intend to use. For	We will use the same method describe in previously published economic evaluations that have used the ACE-Obesity Policy model Ref 1, Ref 2. The modelling involves the following steps:

example, how will total energy sold and energy intake be estimated from your study data and then transformed into BMI for the modelling?	• Estimating the population impacted: A component of the customer survey (described under section 3.4.3) will inform the proportion of individuals attending the facility who change their purchasing behaviour as a result of the intervention, specifically those who substituted unhealthy options with healthier alternatives. The survey also identifies the proportion of consumers who substituted with similar unhealthy products external to the facility. These proportions will inform the size of the population who benefit from the Intervention.<br>• Translating energy sold to energy consumed: The change in energy sold will be converted into changes in daily energy intake, informed by purchasing frequency data obtained from the survey and supplemented by literature-based estimates. Certain assumptions will be applied depending on results of literature search.<br>• Converting energy intake to change in body weight: Changes in energy intake will be translated into changes in body weight using the validated estimate that a sustained change of 100 kilojoules per day corresponds to a 1 kg change in body weight over 3 years[13].<br>• Estimating changes in BMI: Weight changes will be converted to BMI changes using average Australian height and weight data stratified by gender and age groups.
If the modelling will extrapolate impacts at the Victoria State population level, then presumably this means that you would need to model the costs of implementing the intervention at scale as well (i.e. beyond the confines of the hybrid trial). Again, please clarify your intensions here.	We thank the reviewer for this important observation. We confirm that, in addition to trial-based costing, the scaled-up implementation will be modelled and will include cost components associated with broader program management and coordination across Victoria. These costs will be informed by both the trial and relevant literature to reflect implementation at scale.
Will impact of the intervention on overall (total) sales revenues from the food outlets as measured through your sales data be factored into the economic evaluation? Again, please clarify.	While we are collecting sales data, changes in total sales revenue for participating facilities will not be included in the intervention cost or benefit estimates but will be reported separately. The economic evaluation will be

	conducted from a limited societal perspective with the assumption that any changes in revenue/sales within a facility will be redistributed to unimpacted sectors and products. This assumption has been used in previous cost-benefit analyses undertaken for government decision-making Ref.
In relation to the above point, it wasn't clear whether or to what extent the study would measure unintended consequences of the intervention that may be relevant to an economic assessment. For example, if a higher % of sales were for green category products but total sales revenue had fallen, this may be because some people had substituted towards other nearby food outlets selling more unhealthy items (fast food outlets or whatever).	We aim to collect data on substitution intentions in the customer survey (see lines 95-100 for details). We will use this data to factor substitution behaviour into our economic analysis (see lines 183-190 for details).<br><br>Unintended consequences identified in the process evaluation will be incorporated where possible and described qualitatively if these are unable to be quantified and valued. (Lines 217-219)

Additionally, please note that we have included clarifying statements mentioning that the research team will also be collecting data independently (at baseline T0 and a final data collection time point T6) to ensure objectivity and credibility of results for overall trial evaluation. Fig 2 has also been updated to reflect this additional time point of data collection within the intervention group to capture that any final healthy choices implementation changes administered between T5 and trial end will be captured at time point T6. This has been noted in the main manuscript, see lines 304-307.

VERSION 2 - REVIEW

**Reviewer**	**1**
**Name**	**Chan, Sze Ling**
**Affiliation**	**Singhealth, Health Services Research Centre**
**Date**	**27-Jan-2026**
**COI**	

All my comments have been addressed.

**Reviewer**	**2**
**Name**	**Healey, Andy**
**Affiliation**	**King's College London, IOPPN**
**Date**	**02-Feb-2026**

COI

No further comments, thank you.
